# Bibliometric analysis of research hotspots and emerging trends in mitophagy and atherosclerosis (2004–2024)

**DOI:** 10.3389/fmed.2025.1621079

**Published:** 2025-07-25

**Authors:** Shuchen Ding, He Zhang, Luxia Song, Xinyi Wang, Lifang Song, Wende Tian, Xuanye Chen, Hao Xu

**Affiliations:** ^1^Graduate School, Beijing University of Chinese Medicine, Beijing, China; ^2^National Clinical Research Center for Chinese Medicine Cardiology, Xiyuan Hospital, China Academy of Chinese Medical Sciences, Beijing, China; ^3^Hohhot Traditional Chinese and Mongolian Medicine Hospital, Hohhot, China

**Keywords:** atherosclerosis, mitophagy, molecules, pathological process, bibliometrics

## Abstract

**Background:**

Mitophagy is closely involved in the onset, progression and pathological mechanisms of atherosclerosis. This study set out to provide a comprehensive overview and identify emerging research trends in the field.

**Methods:**

A systematic literature retrieval was conducted across the Web of Science Core Collection (WoSCC) for publications spanning 2004 to 2024. Bibliometric analyses and knowledge mapping were performed utilizing CiteSpace, VOSviewer, R-Bibliometrix, Scimago Graphica and Excel to evaluate the intellectual landscape of the field.

**Results:**

The analysis reveals a fluctuating but overall increasing trend in annual publications. The United States and China are the primary contributors to the body of research, with leading institutions predominantly located in China, the United States, and Russia. Notably, the works of Orekhov AN stand out in terms of both quantity and quality. The most cited studies is Forrester SJ’s 2018 publication in *Circulation Research*. Additionally, keyword analysis highlights the prevailing research hotspots, including: (1) key molecules such as NF κB, NLRP3 inflammasome, and mitochondrial DNA; (2) critical pathological processes such as oxidative stress, mitochondrial dysfunction, and mitochondrial dynamics; and (3) and the role of mitophagy within vascular smooth muscle cells, endothelial cells, and macrophages in the pathogenesis of atherosclerosis.

**Conclusion:**

The study of mitophagy in atherosclerosis has garnered increasing attention, with substantial progress made in understanding its molecular and cellular mechanisms. This work highlights the current research hotspots and identifies prospective directions for future exploration. Further investigation into the intricate mechanisms governing mitophagy may uncover novel therapeutic strategies that could mitigate the progression of atherosclerosis.

## 1 Introduction

Atherosclerosis (AS) is a chronic inflammatory disease characterized by lipid metabolic disorders and plaque buildup in the arteries, potentially contributing to cardio-cerebrovascular diseases, such as coronary artery disease, stroke and sudden death (1). As a leading cause of mortality worldwide, AS is driven by global economic growth, harmful dietary patterns, and environmental pollution (2). Its pathogenesis involves a complex, polyfactorial inflammatory process where vascular endothelial cells (ECs), vascular smooth muscle cells (VSMCs), and macrophages play pivotal roles (3). Extensive research has explored the underlying mechanisms of AS. Statins, which reduce low density lipoprotein cholesterol (LDL-C) concentrations, have been considered as the foundation of atherosclerotic cardiovascular disease treatment (4). However, even with LDL-C levels <70 mg/dL, 54–61% residual cardiovascular risk remains, indicating limitations of current lipid-lowering therapies (5). Thus, deeper insights into AS pathogenesis and development of novel therapeutics are essential.

Mitochondria are commonly referred to as the “energy powerhouses of cells.” Identified as the major sources of excessive reactive oxygen species (ROS), impaired mitochondria trigger oxidative stress, which is a critical pro-atherogenic mechanism (6, 7). Mitophagy, the selective removal of dysfunctional mitochondria, is crucial for mitigating ROS production, preventing oxidative stress and maintaining intracellular homeostasis (8). When mitophagy is dysregulated, impaired mitochondria accumulate, exacerbating inflammation and promoting the destabilization of vulnerable AS plaques (9, 10). Indeed, proper mitophagy is vital for the function of key cells in AS development (11, 12). For instance, defective mitophagy may inflict harm on ECs and drive the proliferation and phenotypic transformation of VSMCs (13). A substantial body of research has investigated the association between AS and mitochondrial dysfunction (14), with growing evidence emphasizing the critical role in AS pathogenesis, progression and underlying mechanisms.

Bibliometrics, a mathematical and statistical approach analyzing published papers and its associated data, delineates research landscapes, evaluates academic influence, and identifies emerging trends. This is significant for advancing knowledge in the respective domain. While widely applied across disciplines, bibliometric analysis has not been performed in mitophagy and AS. This study employed CiteSpace and VOSviewer to visualize annual publication trends, countries/institutions, journals, authors, keyword co-occurrences, and co-citation patterns in this field. By analyzing pivotal documents and citation/keyword bursts, we identified key research hotspots and emerging trends, offering valuable insights and addressing current challenges and obstacles of the area.

## 2 Materials and methods

### 2.1 Search strategy

**Figure 1:**
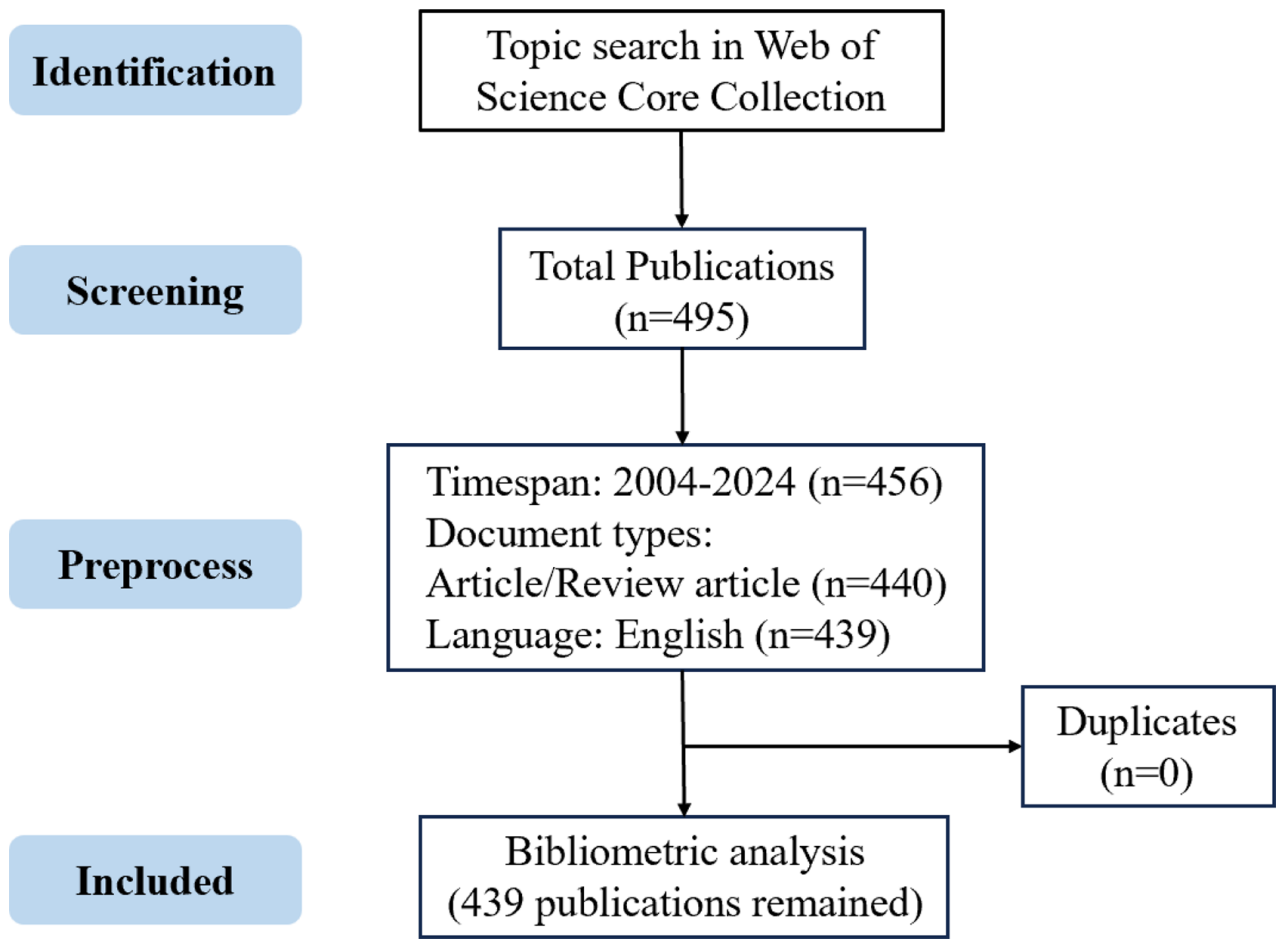
Publication screening flowchart.

The publications on mitophagy and atherosclerosis were retrieved from the WoSCC database. The search string was: TS = (Mitophag OR Mitochondrial autophag OR Mitochondrial degradat* OR Mitochondrial quality control OR Mitochondrial clearanc* OR Mitochondrial remov* OR Clearanc* of mitochondri* OR Remov* of mitochondri* OR Mitochondria-associated degradat*) AND TS = (Arterioscler* OR Fibroatherom* OR Arterial Fatty Streak* OR Atherosclerotic Plaqu* OR Atherom* OR Atheromatous Plaqu*)**. The search scope included titles, abstracts, and keywords. The specific retrieval strategy was presented in Supplementary Table 1. The resulting records contained 495 documents. All documents published between 2004 and 2024 were retrieved on July 9, 2025. Articles and reviews published in English were included in our research. After removing duplicates, there were finally 439 publications that met the criteria to conduct bibliometric analysis and visualization. The detailed flowchart depicting the publication screening process is presented in Figure 1.

### 2.2 Ethical consent

No human or animal subjects were involved in this study.

### 2.3 Data analysis

The information of 439 publications selected include: title, authors, countries, institutions, journals, publication year, keywords and reference records, etc. The above collected data was imported into mainstream bibliometric software, including CiteSpace (version 6.4. R1), VOSviewer (version 1.6.20), the R-Bibliometrix 4.1.0 package and R-studio to carry out visualization and consequently comprehensive analysis. Besides, Microsoft Excel 2016 was applied to draw radar maps, histograms and line charts. By utilizing such software, we managed to extract crucial information from enormous literatures and establish visual maps which furnished perspectives on the research.

## 3 Results

### 3.1 Global overview

**Figure 2:**
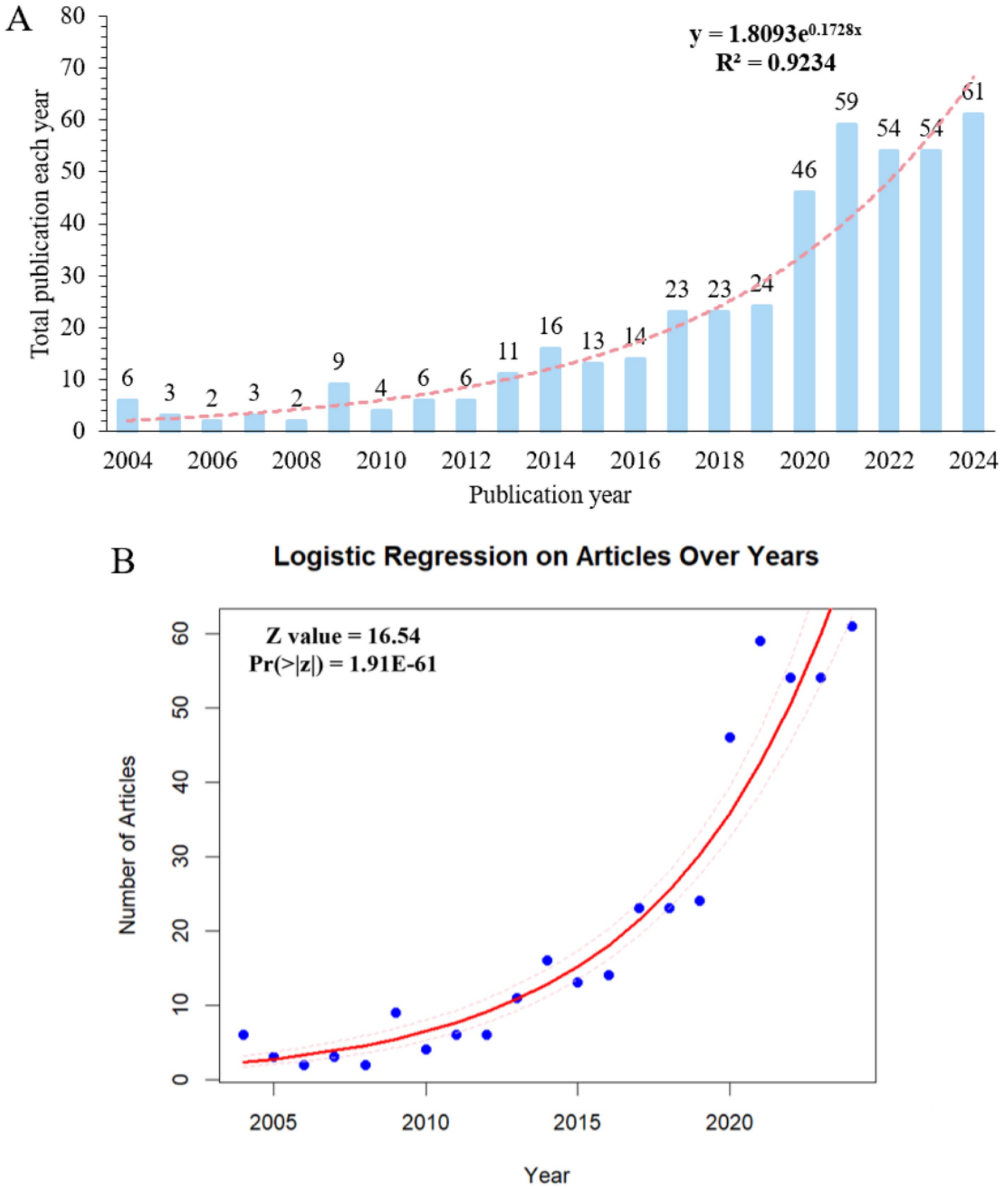
**(A)** Annual output of mitophagy in atherosclerosis publications from 2004 to 2024. **(B)** Logistic regression on number of articles over years.

A total of 439 publications were published in the area of mitophagy and atherosclerosis from 2004 to 2024. The annual growth of publications presents a rising trend in Figure 2A. Specifically, the annual volume of literature in this area was fewer than nine publications per year prior to 2012. From 2013 to 2019, there was limited growth in this field, with less than 24 papers published yearly yet. It grew rapidly from 2020 onward, and the annual outputs fluctuated between 46 and 61, showing a strikingly greater focus on mitophagy and atherosclerosis. Microsoft Excel 2019 was applied to analyze the trend of publications per year. The fitted equation was y = 1.8093e^0.1728x^ and R^2^ = 0.9234, demonstrating a well-fitted nature. Logistic regression analysis of the trend in the number of articles over years is presented in Figure 2B, and prediction intervals are displayed in Supplementary Table 2. In general, the volume of publications per year in the area of mitophagy and atherosclerosis has been in an overall uptrend.

### 3.2 Countries/regions and institutions distribution

**Figure 3:**
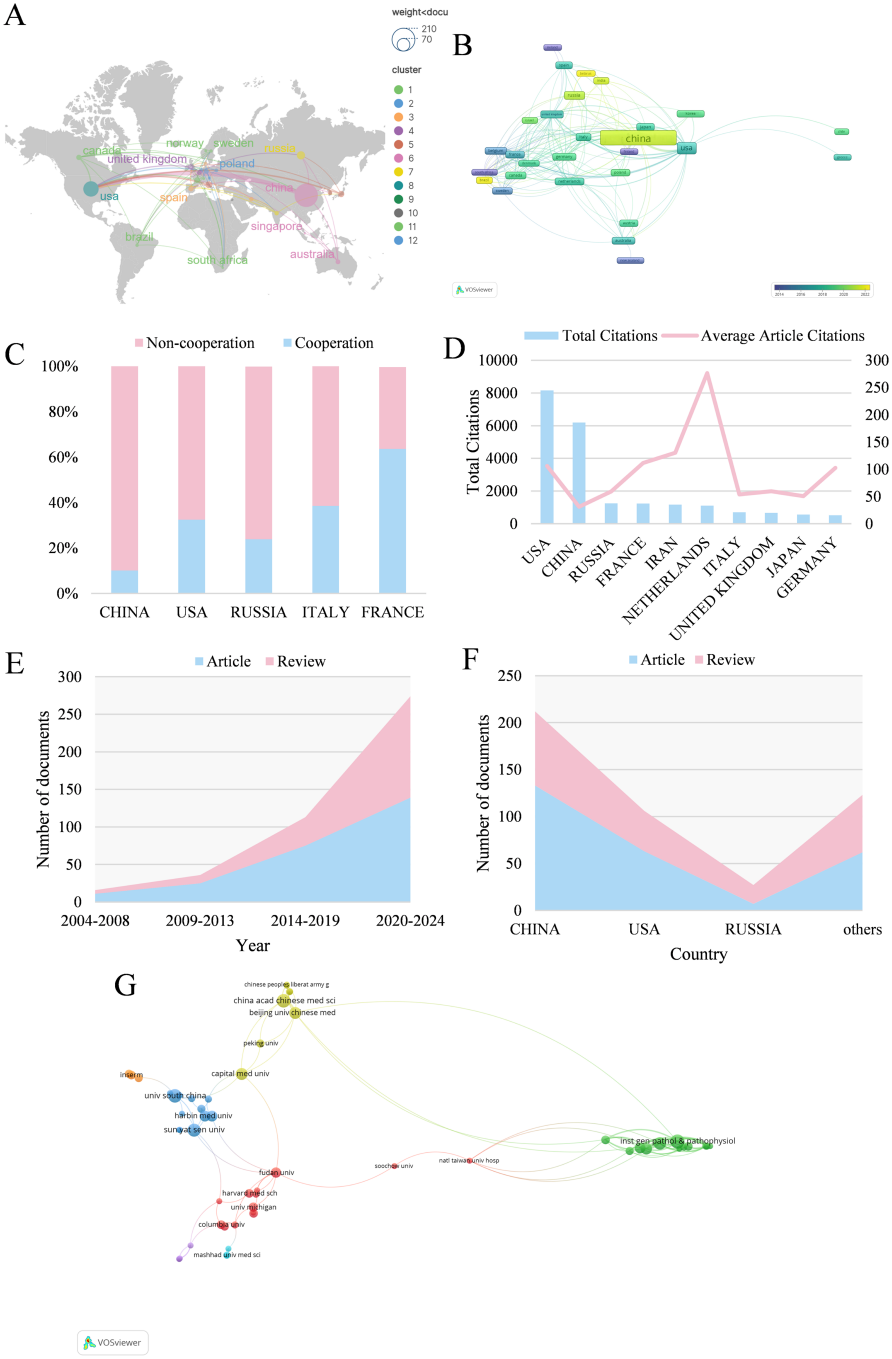
**(A)** The geographical distribution in terms of publications. The larger circle indicates a higher number of articles, and the cooperation is exhibited as links between nodes. **(B)** The co-occurrence network map of countries/regions. Each node represents a country, where the size of the node indicates the publication volume. **(C)** Proportional distinction of national cooperation among different countries. **(D)** The number of total and average citations of the top ten productive countries. **(E)** The growth curve of different years. **(F)** The proportion of articles and reviews among countries. **(G)** Institutions co-occurrence map.

Based on the publications collected (Table 1), China tops the list with the highest publication output (199, 45.33%), followed by the USA (77, 17.54%) and Russia (21, 4.78%). The distribution of national production and the co-occurrence network of countries/regions are, respectively, shown in Figures 3A,B. The USA (0.56) has the highest betweenness centrality, followed by United Kingdom (0.28) and Russia (0.09), indicating their roles as key connectors in the international collaboration network. In terms of the top five countries with the largest publication volume, 64.00% of the studies published in France are completed in cooperation with other countries, while collaborative articles published in China account for only 10.10% (Figure 3C). Additionally, the USA (8152) and China (6190) contribute most to the total citations (Figure 3D), which greatly outnumbered the others. There has been rapid increase for recent years in both experimental articles and review literatures (Figure 3E). Regarding documents published in China and the USA, the volume of experimental studies is far higher than that of review articles (Figure 3F). On the whole, China and the USA are the main countries/regions contributed to the field of mitophagy and AS, with the most publications and the greatest influence.

**Table 1:** Top 10 most productive countries/regions.

Rank	Country	Documents	Percentage	Betweenness centrality
1	China	199	45.33	0.07
2	USA	77	17.54	0.56
3	Russia	21	4.78	0.09
4	Italy	13	2.96	0.04
5	France	11	2.51	0.04
6	Japan	11	2.51	0.03
7	United Kingdom	11	2.51	0.28
8	Iran	9	2.05	0.08
9	Canada	8	1.82	0.03
10	Korea	7	1.59	0.00

A total of 362 institutions are included. Among major research institutions, Institute of General Pathology and Pathophysiology (20 papers) and Russian Academy of Medical Sciences (20 papers) leads in publication count, followed by China Academy of Chinese Medical Sciences (15 papers) and University of South China (14 papers) (Table 2). Among the top ten most prolific institutions, five of which are located in China. Some of these institutions exhibits a betweenness centrality value lower than 0.01, suggesting sparse collaboration and the need to enhance partnerships with other institutions. Institution co-occurrence map is shown in Figure 3G.

**Table 2:** Top 10 most productive institutions.

Rank	Institution	Country	Documents	Betweenness centrality
1	Institute of General Pathology and Pathophysiology, Rams	Russia	20	0.01
2	Russian Academy of Medical Sciences	Russia	20	0.01
3	China Academy of Chinese Medical Sciences	China	15	0.02
4	University of South China	China	14	0.03
5	National Medical Research Center of Cardiology	USA	13	0
6	Research Institute of Human Morphology	Russia	13	0.01
7	Sun Yat-sen University	China	13	0
8	Beijing University of Chinese Medicine	China	11	0.04
9	Capital Medical University	China	11	0.06
10	Russian Academy of Sciences	Russia	11	0

### 3.3 Journals and co-cited journals

**Figure 4:**
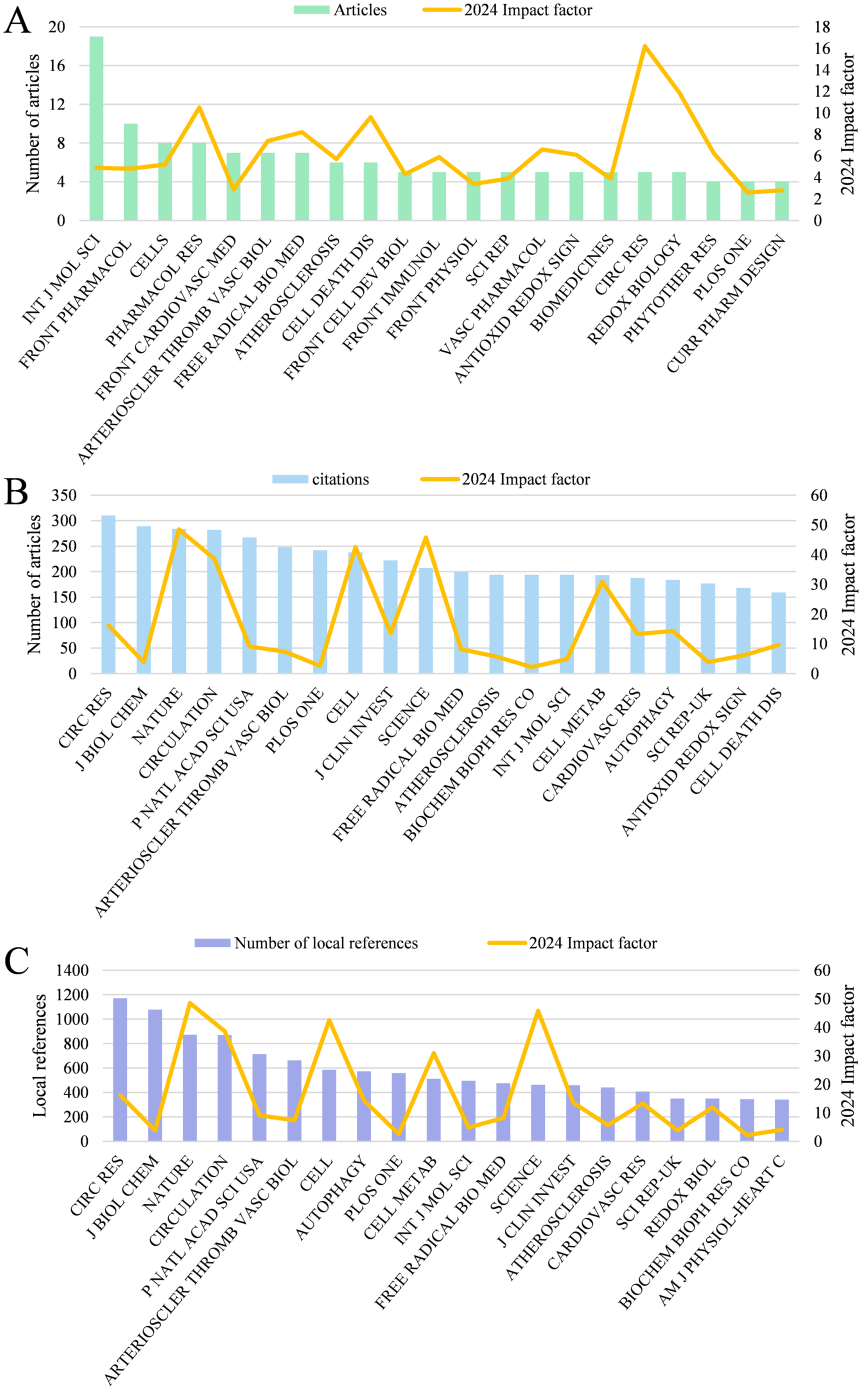
**(A)** The top 20 journals with the highest output quantity and their impact factor. **(B)** The top 20 co-cited journals. **(C)** The top 20 journals with the highest number of local references and their impact factor.

439 papers were published on 243 journals. Figure 4A shows the top 20 journals in terms of publication volume in the field of mitophagy and AS, the top 4 of which were “International Journal of molecular sciences,” “Frontiers in Pharmacology,” “Cells,” and “Pharmacological Research,” with 19, 10, 8, 8 articles, respectively. It is worth mentioning that 75% of the top 20 were categorized as Q1 in Journal Citation Reports (JCR), five journals had an impact factor exceeding 8, with “Circulation Research” (IF = 16.2) and “Redox Biology” (IF = 11.9) in the lead, which represents a high research significance in mitophagy and AS. “Circulation Research” (310) is the most co-cited journal, followed by “Journal of Biological Chemistry” (289), “Nature” (284), “Circulation” (282) and “Proceedings of the National Academy of Sciences” (267) (Figure 4B). These journals act as critical sources of knowledge, highlighting their essential role in this area. The top five journals of local cited articles in this research field are: “Circulation Research,” “Journal of Biological Chemistry,” “Nature,” “Circulation” and “Proceedings of the National Academy of Sciences” (Figure 4C). The top 20 journals incorporate a number of top journals on the subject, such as “Nature,” “Cell,” “Science,” “Circulation,” etc. This embodies the solid theoretical underpinnings of mitophagy and atherosclerosis.

### 3.4 Authors and co-cited authors

**Figure 5:**
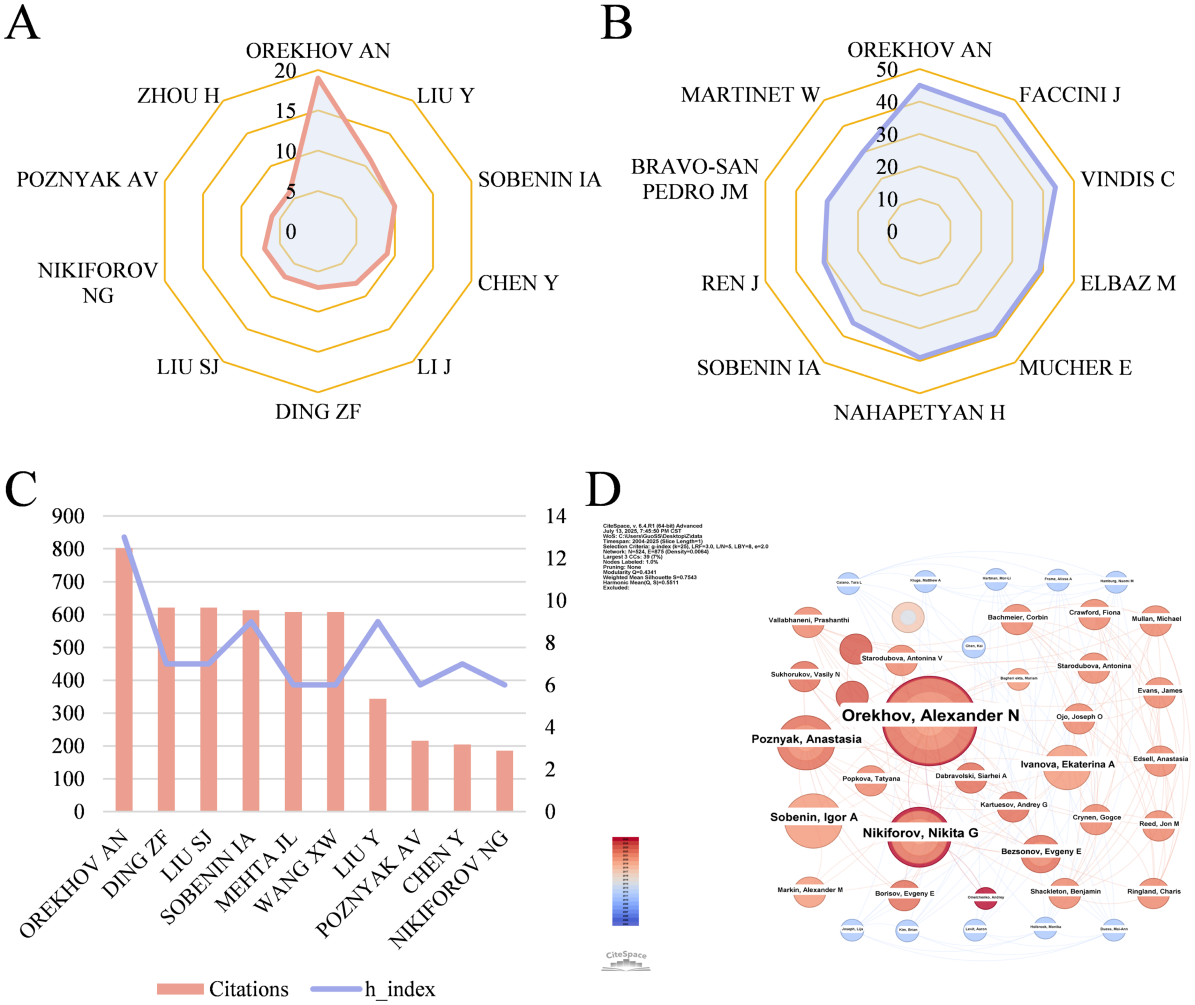
**(A)** The top 10 authors with the highest output quantity. **(B)** The top 10 authors with the highest number of local citations. **(C)** The top 10 authors in terms of citations and their H index. **(D)** Author co-occurrence map in mitophagy of AS. Each node represents an author, where the size of the node indicates the publication volume and different colors of circles suggest the publication year.

2,640 authors in total participated in the study of mitophagy in atherosclerosis. Among them, the top 3 most productive authors are Orekhov AN (19 papers), Liu Y (11 papers) and Sobenin IA (10 papers) (Figure 5A). Interestingly, Orekhov AN and Sobenin IA both come from Russia. The top 3 authors with the highest local citations are Orekhov AN (45), Faccini J (44) and Vindis C (44) (Figure 5B). Besides, the top 3 authors with the highest citations are Orekhov AN (802), Ding ZF (621) and Liu SJ (621) (Figure 5C). We calculated the H index to estimate the quantity and quality of researches by scholars. In terms of H index, Orekhov AN (13), Sobenin IA (9), Liu Y (9) take a lead.

Orekhov AN, from Institute of General Pathology & Pathophysiology in Russia, ranked first in the list of highest output, highest citations and highest H index, which reflected his exceptional contributions and influence. The author co-occurrence map is shown in Figure 5D, displaying an extensive cooperation of authors with several scattered collaborative networks. Nonetheless, no author has a betweenness centrality over 0.01.

### 3.5 Co-cited references and burst references

**Figure 6:**
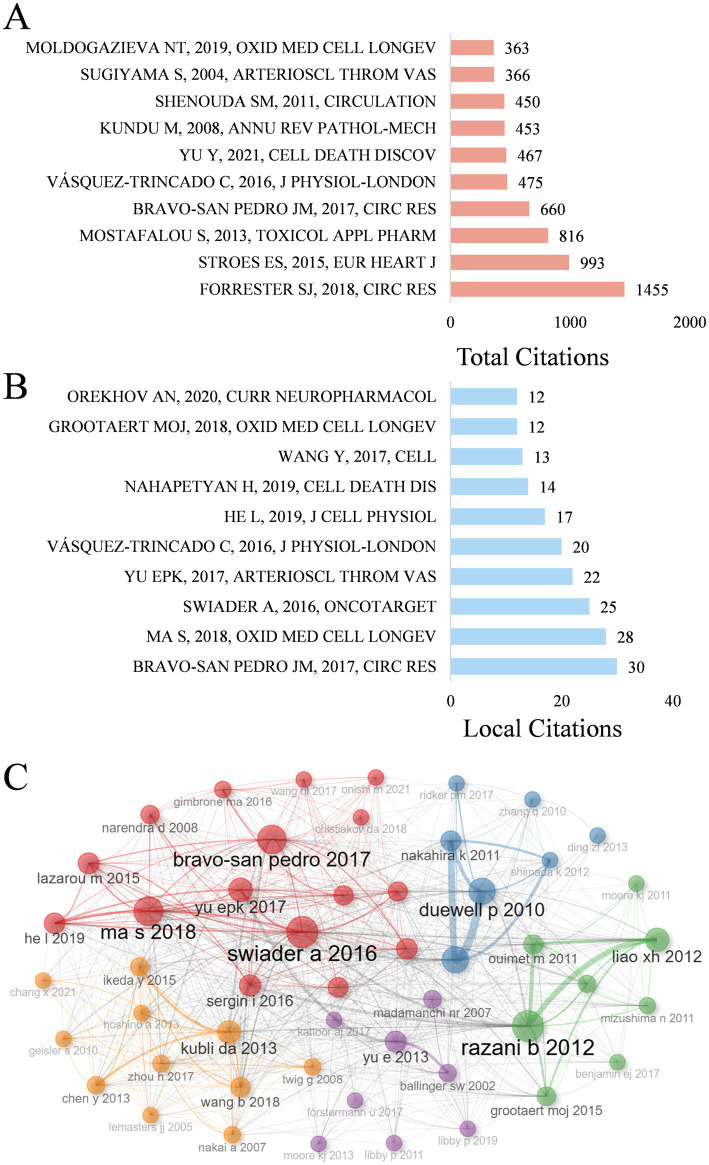
**(A)** The top 10 most cited by global literature. **(B)** The top 10 local cited papers. **(C)** Co-citation network of key papers.

The function and mechanisms of mitophagy in atherosclerosis has attracted extensive attention and citations. Global citations (GCS) and local citations (LCS) were applied to appraise cited articles, and the top 10 papers with the highest GCS and LCS were presented in Figures 6A,B, manifesting a higher recognition by experts on the subject. A review article written by Forrester SJ published on Circulation Research in 2018 possesses the highest total citations (*n* = 1,455), which provided a comprehensive and in-depth understanding for the role of ROS in metabolic and inflammatory signaling pathways, indicating its fundamental role in related field (15). Another paper completed by Bravo-San Pedro JM on Circulation Research in 2017 ranks first in local citations (*n* = 30), which also occupies the fourth place of the list of total citations (*n* = 660). They reviewed the function of autophagy and mitophagy in safeguarding cardiovascular homeostasis, along with their pathological mechanisms under pathological circumstances (16). In addition, a cross-citation network among these key papers was depicted in Figure 6C.

**Figure 7:**
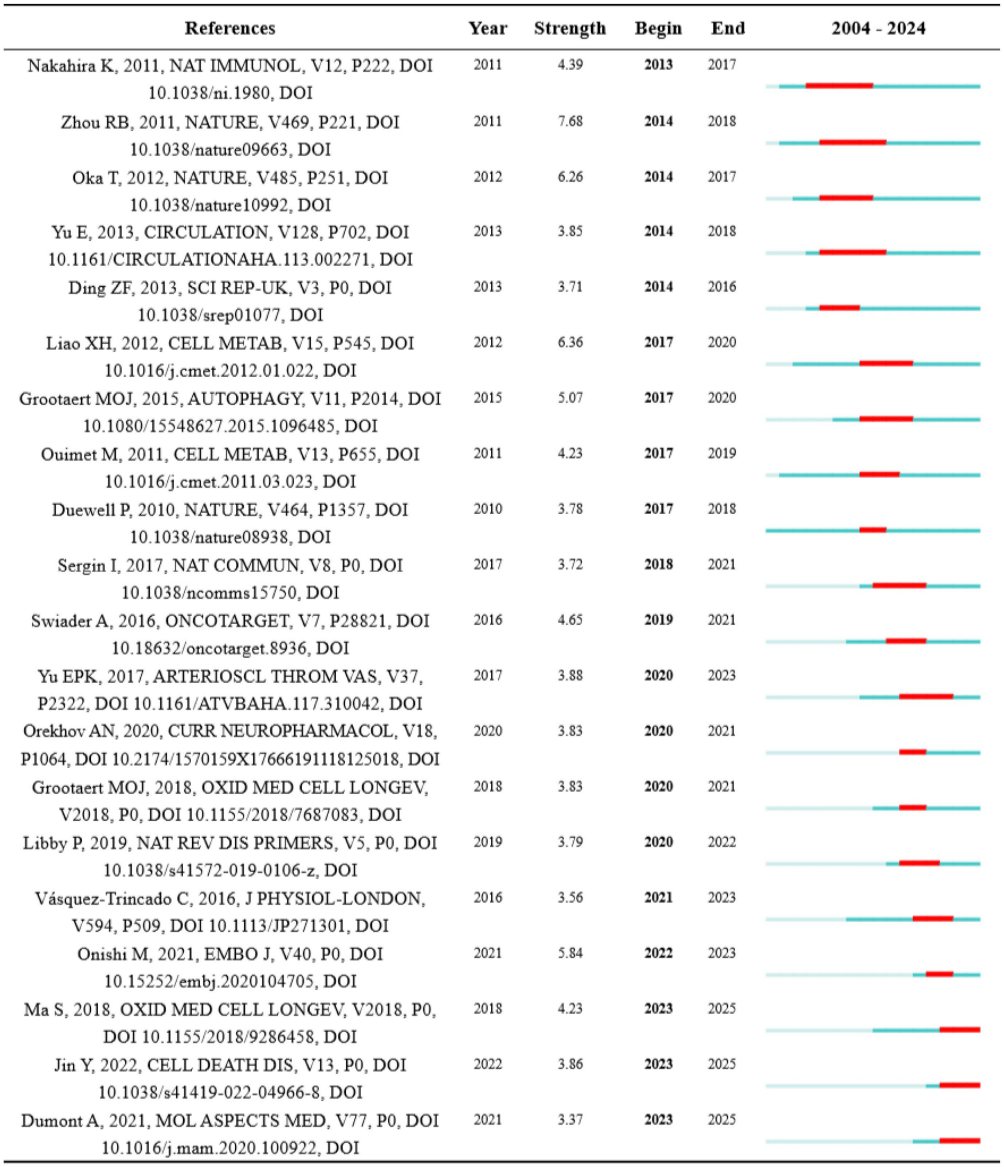
Top 20 references with the strongest citation burst. Strength indicates the intensity of the burst, the blue bars represent the paper published initially, the red bars mean citation burst.

Citation bursts in references represent a frequency surge in citation counts across time, reflecting their rapidly widespread recognition and dissemination within the research domain. Figure 7 displays the 20 references exhibiting the highest citation burst. A letter exhibited the strongest burst signal in citations, which is published by Zhou RB on Nature in 2011 (17), with citation burst from 2014 to 2018. Notably, 3 references are still in strong burst. Sai Ma conducted an animal experiment to investigate the function and mechanisms of melatonin ameliorating the development of AS through the activation of mitophagy and inhibition of NOD-like receptor family pyrin domain containing 3 (NLRP3) inflammasome (18). Ying Jin assigned an innovative role of caspase 1 inhibitor VX765 in restraining the assembly of NLRP3 inflammasome, as well as inhibiting AS by facilitating mitophagy and efferocytosis (19). Adelie Dumont reviewed the role of mitochondria to modulate macrophage effector functions in AS (20).

### 3.6 Keywords analysis and potential research hotspots

**Figure 8:**
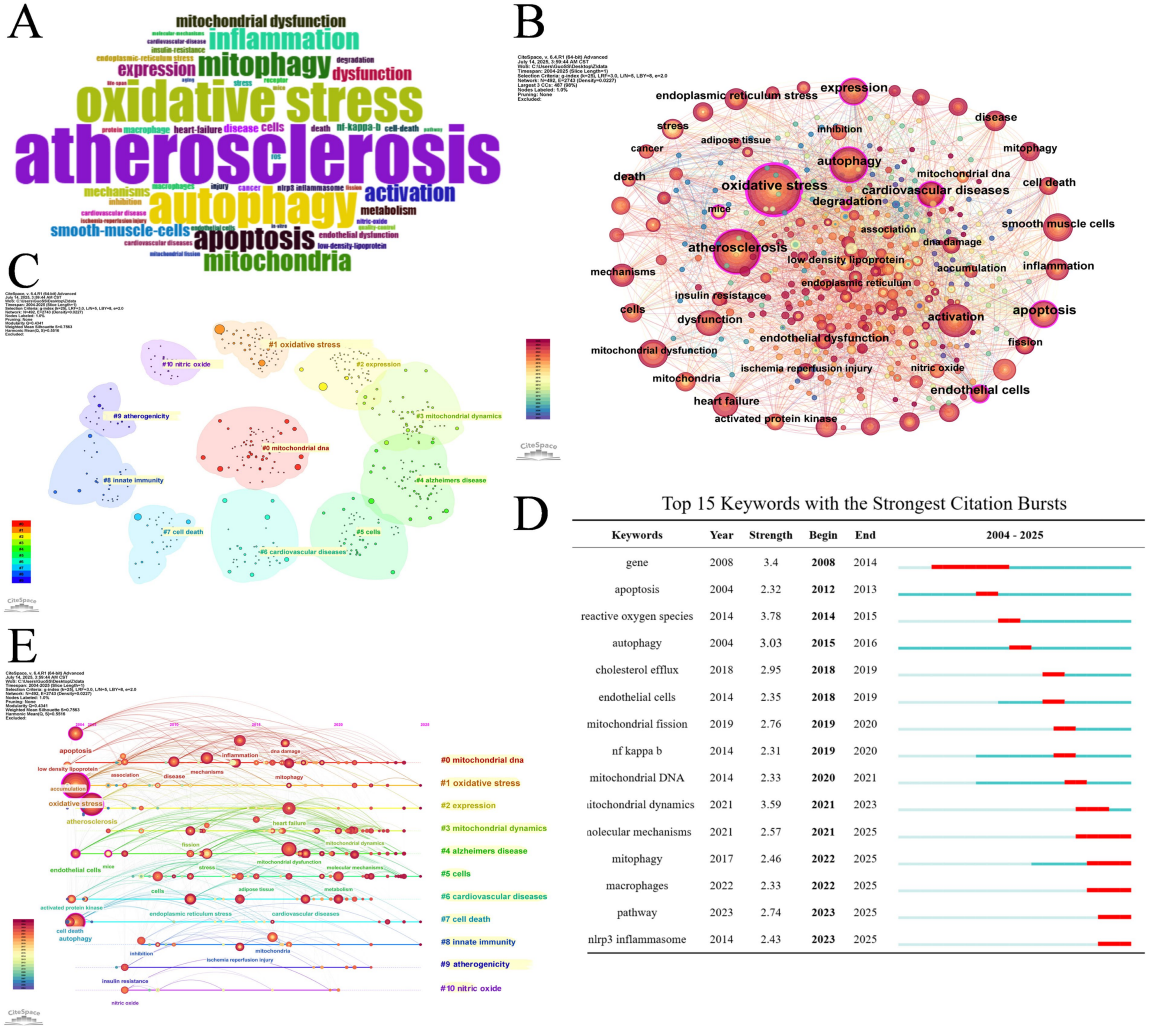
**(A)** Word cloud of keywords. **(B)** Keyword co-occurrence network map. **(C)** Cluster analysis of keywords. **(D)** Top 15 keywords with the strongest citation bursts. **(E)** Timeline distribution of the top 9 clusters.

The cloud map of keywords (Figure 8A) and keyword co-occurrence map (Figure 8B) display that oxidative stress, AS, autophagy, activation, apoptosis, expression, dysfunction, mitochondrial dysfunction and smooth muscle cells are high-frequency keywords. By cluster analysis based on keywords, 11 clusters were exhibited in Figure 8C, including: #0 mitochondrial DNA, #1 oxidative stress, #2 expression, #3 mitochondrial dynamics, #4 Alzheimer’s disease, #5 cells, #6 cardiovascular diseases, #7 cell death, #8 innate immunity, #9 atherogenicity, #10 nitric oxide. Moreover, Figure 8E displays the keyword timeline distribution, intuitively showing the progression of keywords in each cluster on its timeline.

Table 3 presents the ten most frequent keywords for molecules, pathological processes and disease correlated with mitophagy in AS. It shows that nuclear factor kappa B (NF κB) (*n* = 26), NLRP3 inflammasome (21), mitochondrial DNA (19), nitric oxide (17), low density lipoprotein (13), ROS (13), parkin (10) are the most explored molecules; oxidative stress (149), mitochondrial dysfunction (87), apoptosis (68), activation (61), mitochondrial dynamics (51) are the most studied pathological process; cardiovascular diseases (57), heart failure (28), myocardial infarction (16), coronary artery disease (14), Alzheimer’s disease (12) are the most mentioned diseases in mitophagy in AS.

**Table 3:** Top 10 most frequent keywords for molecules, pathological process and disease correlated with mitophagy and AS.

Molecules	Counts	Pathological process	Counts	Diseases	Counts
NF κB	26	Oxidative stress	149	Cardiovascular diseases	57
NLRP3 inflammasome	21	Mitochondrial dysfunction	87	Heart failure	28
Mitochondrial DNA	19	Apoptosis	68	Myocardial infarction	16
Nitric oxide	17	Activation	61	Coronary artery disease	14
Low density lipoprotein	13	Mitochondrial dynamics	51	Alzheimer’s disease	12
ROS	13	Smooth muscle cells	48	Cancer	11
Parkin	10	Expression	47	Diabetic cardiomyopathy	7
Angiotensin ii	7	Endoplasmic reticulum stress	36	Cardiac hypertrophy	5
Mitofusin 2	7	Endothelial cells	36	Diabetes mellitus	4
Kinase	7	Inflammation	29	Hypertension	3

Burst keywords refer to those undergo a sudden increase in utilization within a period of time. The 15 strongest burst keywords are listed in Figure 8D chronologically. Before 2021, ROS, ECs, cholesterol efflux, NF κB, mitochondrial DNA and mitochondrial dynamics were hot research topics involved in the mechanism of mitophagy and AS. Words include molecular mechanisms, mitophagy, macrophages, pathway and NLRP3 inflammasome surged after 2021, demonstrating that these topics may be the research hotspots for the present and future.

## 4 Discussion

### 4.1 General information

In this study, a bibliometric analysis of mitophagy and AS was carried out to obtain insights into the research hotspots and popularity trends within the domain. As far as we know, this is the first bibliometric analysis focused specifically on mitophagy and AS. It addresses previous gaps in understanding the role of mitophagy in AS and highlights potential opportunities for collaboration and future research directions, thereby providing valuable guidance for subsequent studies in this area.

Over the past two decades, there has been a notable increase in the volume of publications on mitophagy and AS. Initially, this emerging concept garnered little attention. Research on this topic before 2012 was surprisingly limited, with no more than nine papers published per year. However, the output gradually increased over the following 8 years, reaching around 20 in articles annually. A significant turning point occurred in 2020, when the number of publications doubled compared to previous years, although the total volume remains modest. This trend is clearly reflected in our analysis, highlighting both the growing interest and evolving perception of this field. The observed annual growth pattern implicates that future research regarding mitophagy and AS holds considerable potential.

Analysis of countries and institutions allows for the identification of regions and organizations exerting significant influence, as well as their collaborative efforts in the domain of mitophagy and AS. It is presented that China takes the lead in terms of publication volume, while the USA ranks first in total citations, indicating the substantial contributions of both countries in advancing research on mitophagy in AS. The publication output and citation count of these two nations far surpass those of others. Additionally, countries such as Russia and France have made notable contributions to the development of this area. Among the top 10 most prolific institutions, five are from China, four from Russia and one from the USA. The leading institutions based on publication volume include Institute of General Pathology and Pathophysiology, Russian Academy of Medical Sciences, China Academy OF Chinese Medical Sciences and University of South China. Concerning international cooperation, France and the USA have formed partnerships with multiple countries, underscoring the extensive global impact and great potential of research on mitophagy and AS.

The top 20 journals in terms of publication volume in the field of mitophagy and AS include both comprehensive journals and specialized publications in areas such as immunology and cardiovascular research. Among these, three journals exhibit an impact factor (IF) greater than 10, while 11 journals possess an IF above 5. Journals with an IF above 10 are “Circulation Research” (IF = 16.2), “Redox Biology” (IF = 11.9) and “Pharmacological Research” (IF = 10.5). The four journals with the highest publication volume are “International Journal of molecular sciences,” “Frontiers in Pharmacology,” “Cells” and “Pharmacological Research.” Prominent journals with the highest co-citation rates are “Circulation Research,” “Journal of Biological Chemistry,” “Nature,” “Circulation” and “Proceedings of the National Academy of Sciences.” Generally, these journals are expected to serve as core outlets for publishing articles and research findings in the field of mitophagy and AS.

Orekhov AN, an expert from Institute of General Pathology & Pathophysiology in Russia, tops the list of highest output, highest citations and highest H index, reflecting his exceptional contributions and influence on this topic. His research has primarily focused on inflammation, mitochondrial function and AS. Recent articles by his team have provided in-depth insights into mechanisms such as mitochondrial function and mitochondrial gene mutations in health and diseases, particularly in the context of AS. Sobenin IA, from National Medical Research Center of Cardiology in Russia, ranks third in terms of publication volume. His research has centered on the mechanisms underlying AS, with a focus on lipids, mitochondrial function and immunology. Nikiforo NG, another prominent researcher from Institute of General Pathology & Pathophysiology in Russia, is likewise recognized as one of the prolific authors in related area. These three Russian researchers have established extensive collaboration, contributing significantly to the advancement of knowledge in mitophagy and AS.

Analysis of key articles is essential for grasping the foundational knowledge and advancing research in the field. Forrester SJ et al. provided a comprehensive review of the critical role of ROS in metabolic and inflammatory signaling pathways, emphasizing their pathological mechanisms in cardiovascular and metabolic diseases, such as atherosclerosis (15). ROS contributed to disease progression not only by triggering inflammatory signaling pathways (e.g., NF κB, NLRP3) but also by modulating mitochondrial function and autophagy, thereby influencing cellular metabolic homeostasis. This article remains the most cited publication (*n* = 1,455) in total. Bravo-San Pedro JM’s work, cited 660 times in total and 30 times as the most locally cited, reviewed the protective function of autophagy and mitophagy in maintaining cardiovascular homeostasis, as well as their pathogenic mechanisms under pathological conditions, highlighting their potential therapeutic value (16). This study demonstrated that mitophagy, by eliminating damaged mitochondria that generate ROS, plays a protective role in cardiomyocytes. Furthermore, genetic defects in mitophagy-related proteins, such as PARK2 or PINK1, gave rise to increased cytotoxicity in VSMCs exposed to oxidized LDL (oxLDL).

Kundu M et al. also investigated the pivotal role of autophagy in maintaining cellular health and regulating pathological conditions, and its relevance to various disease including atherosclerosis (21). Vásquez-Trincado C explored the critical function of mitochondrial dynamics, including mitophagy, in cardiovascular health and disease, demonstrating that dysfunction in these processes brought about the progression of cardiovascular pathologies like atherosclerosis (22). In addition, Yu Y et al. summarized the link between ferroptosis, oxidative stress, and inflammation in cardiovascular diseases (23). Ferroptosis, featured with iron overload and lipid peroxidation (LPO), triggered ROS production via Fenton reaction. The resulting excess ROS, including mitoROS, further promotes oxidative stress, creating a vicious cycle that exacerbates LPO production and inflammatory reaction. Review articles mentioned above are among the top 10 most cited globally or locally, providing a solid theoretical foundation for ongoing research in this area.

### 4.2 Research hotspots and emerging topics

#### 4.2.1 Mechanisms of mitophagy in AS research

NF κB and NLRP3 inflammasome have emerged as some of the strongest burst keywords in recent studies. NF κB, widely recognized as a crucial activator of inflammation induced by ROS, has been substantiated to be associated with atherosclerosis through enhanced inflammatory responses, endothelial dysfunction and vascular remodeling. OxLDL conduces to atherogenesis through ROS-dependent mechanisms, where the activation of NF κB results in macrophage cytokine production and cell death. On the other hand, NF κB possess the capacity to limit the excessive activation of the NLRP3 inflammasome, and can promote autophagy by stimulating the expression and activity of autophagy-related proteins in macrophages, which is embodied in the amount of SQSTM1 (24). As mitophagy is enhanced, excessive inflammation and macrophage death caused by mitochondrial damage-associated molecular patterns (mito-DAMPs) are suppressed. Impaired mitochondria are considered significant endogenous damage-associated molecular patterns (DAMPs), including oxidized mtDNA and ROS (25). In the presence of abortive mitophagy, mito-DAMPs trigger excessive inflammatory responses and further pathological changes. Notably, studies have revealed that dihydromyricetin acts as a regulator to facilitate mitophagy in mice, mitigating the activation of NF κB and subsequent NLRP3 inflammasome, thereby offering protection versus atherosclerosis (26). Given that NF κB functions as a double-edged sword, balancing its proinflammatory effects with protective role of mitophagy is crucial to achieving cardiovascular benefits.

Recently, NLRP3 inflammasome, a significant burst keyword in research on mitophagy and AS, has attracted increasing attention since 2023. As a crucial immune sensor of cellular stress signals (27), NLRP3 inflammasome is considered as a key contributor to the progression of AS. Notably, mitochondrial dysfunction is of vital importance for activating NLRP3 inflammasome (28). A study elucidated that VX765, a caspase 1 inhibitor, suppressed the fabrication and activation of NLRP3 inflammasome, ameliorated mitochondrial impairment caused by NLRP3 inflammasome, and facilitated mitophagy, efferocytosis and M2 polarization of macrophages, ultimately playing a part in antagonizing AS (19). In the process, reduced production of mitochondrial ROS and cytosolic release of mtDNA were observed. The article mentioned above manifests strong citation burst since 2023, underscoring the continued focus on NLRP3 inflammasome as a critical area of research within mitophagy and AS. In general, a balanced activation of NLRP3 inflammasome is essential for maintaining cellular and mitochondrial homeostasis. Therefore, further investigation into the precise mechanisms underlying this process is imperative.

Mitochondrial DNA (mtDNA) is a keyword with strong burst since 2020. Orekhov AN and his team have made crucial contributions to understanding the role of mitochondrial DNA in AS. Studies have disclosed that specific mtDNA mutations in the leukocytes of atherosclerotic people are associated with AS (29). Dysfunction of mitochondria has been identified as a cause of innate immune disorders under the circumstances of AS, with certain mutations resulting in mitochondrial dysfunction, thus altering monocyte-derived macrophage activation in AS (30). Remarkably, specific mtDNA mutations have been found to impair mitophagy (31). Orekhov AN proposed a hypothesis to explain the vital role of these mutations in the initiation and progression of AS. When mtDNA mutations impair mitophagy in macrophages, the pro-inflammatory response failed to resolve and instead intensifies with each pro-inflammatory stimulus, leading to chronic inflammation in the vascular wall. This process is further exacerbated by unregulated lipid accumulation, culminating in the formation of atherosclerotic lesions. Another fascinating possibility is that dysfunctional mitochondria could be recognized as pathogens, displaying foreign epitopes that stimulate the immune reaction. In conclusion, damaged mitophagy caused by mtDNA mutations may lead to innate immune disorders and the onset of chronic inflammation. Moreover, deletions and mutations in mtDNA could potentially serve as biomarkers for diseases. Studies have also disclosed that mitigating mtDNA impairment and promoting mitochondrial respiration assist in the decrease of necrotic core and the increase of fibrous cap regions, presenting a promising therapeutic strategy for AS (32).

Mitophagy pathways involved in AS have emerged as a significant area of research. Several mitophagy pathways have been identified, which can be classified into ubiquitin-mediated and receptor-mediated pathways. The PINK1/Parkin pathway is a key component of ubiquitin-mediated mitophagy, while receptor-mediated pathways include BNIP3-mediated mitophagy, FUNDC1-mediated mitophagy, and lipid-mediated mitophagy (13). Other potential pathways, such as AMPK/ULK1-mediated mitophagy, are also under investigation. Among these, the PINK1/Parkin mitophagy pathway has garnered the most attention, whereas research into other pathways remains relatively nascent. Importantly, there is an interaction between these mitophagy pathways and traditional mechanisms underlying atherosclerosis. The same protein may participate in multiple mitophagy pathways and respond to various stresses, exhibiting distinct modes of expression. This complexity underscores the necessity for further research to elucidate the precise role of mitophagy in AS and to facilitate the development of mitochondrial-targeted therapies. Since 2023, the keyword “pathway” has shown a marked burst in scholarly activity, signaling that the specific mechanisms underlying these pathways are still under intense investigation.

#### 4.2.2 Mitophagy in different type of cells in AS

VSMCs, ECs and macrophages are high-frequency and strong-burst keywords respectively, reflecting their pivotal roles in the onset and progression of AS. In recent years, numerous studies have focused on investigating the involvement of mitophagy in VSMCs, ECs and macrophages within the context of AS, and this line of research continues to evolve. Given their critical functions in vascular homeostasis, effectively modulating the activity of VSMCs, ECs and macrophages is crucial for both the prevention and treatment of AS.

A wealth of evidence has illustrated that the apoptosis of VSMCs is closely associated with features of vulnerable plaques in atherosclerosis (33, 34). Swiader A et al. were the first to discover that PINK1/Parkin-mediated mitophagy in response to oxLDL occurred in human VSMCs (35). Silencing PINK1 and Parkin attenuated mitophagy and exacerbated oxLDL-induced vascular smooth muscle cell (VSMC) apoptosis, whereas overexpression of these proteins conferred protective effects via restricting cell death. By using apolipoprotein E-deficient (ApoE−/−) mice with the specific autophagy gene Atg7 deleted in VSMCs, it is enunciated that the loss of autophagic flux in VSMCs impaired mitochondrial quality control, which involved defective mitophagy and biogenesis (36). This dysfunction exacerbated VSMC apoptosis and further plaque vulnerability. These findings suggest that targeting mitochondrial quality control may represent a promising therapy for the stability of atheromatous plaques. However, a contrasting study using human aortic VSMCs, ApoE−/− mice and PINK1−/− mice reached different conclusions. In the context of abnormal VSMC proliferation associated with atherosclerosis (37), it is reported that PINK1/Parkin-dependent mitophagy participated in apelin-13-induced human aortic VSMC proliferation through the enhancement of p-AMPKα, thereby aggravating the progression of atherosclerotic lesions (38). These contradictory findings underscore the need for further investigation into the role of mitophagy in VSMCs and its specific mechanisms in the pathophysiology of AS.

The endothelium maintains vascular homeostasis by adjusting vascular tension and permeability, as well as the proliferation of VSMCs (39, 40). Endothelial dysfunction is a critical contributor to the pathophysiology of atherogenesis. Considered as a major source of ROS, defective mitochondria are implicated in endothelial dysfunction, promoting intravascular thrombosis, leakage and inflammation (41). Shenouda SM et al. observed mitochondrial fragmentation and elevated expression of fission-related proteins in venous ECs from diabetics (42). Under pathological circumstances like diabetes, increased mitochondrial fission and impaired autophagy resulted in the accumulation of dysfunctional mitochondria and heightened mitoROS production, suggesting that increased mitochondrial fission contributed to endothelial dysfunction in diabetic conditions. This study highlights the importance of mitochondrial dynamics, a research topic that has experienced a significant citation burst in recent years. Further studies have revealed that high concentrations of glucose and lipids induce excessive ROS production and impair mitophagy (13). As damaged mitochondria accumulate, endothelial dysfunction and apoptosis ultimately gave rise to atherogenesis. It has been proposed that an imbalance between oxygen supply and demand accelerates the development of AS (43). Intriguingly, some experts hold the idea that promoting mitophagy alleviates hypoxia-induced endothelial dysfunction, with mitochondrial quality control improved and ROS production reduced (44). However, an alternative perspective suggests that suppressing mitophagy ameliorates hypoxia-induced EC dysfunction and inflammation reaction (45, 46). Therefore, the therapeutic benefits of mitophagy promotion in hypoxic conditions remain a matter of debate. Notably, the association between mitophagy activation following hypoxia and AS is surprisingly limited.

It is well-established that macrophages function differently based on their phenotypic polarization. M2 macrophages are involved in anti-inflammation reaction, tissue regeneration and wound recovery, while M1 macrophages are implicated in pro-inflammatory responses and plaque disruption (47). A study has disclosed that mitochondria-located apolipoprotein A-I binding protein (AIBP) may help counteract AS probably via modulating PINK1-induced mitophagy and M1/M2 polarization (48). Specifically, mitochondrial AIBP has been shown to enhance mitophagy and engage in mitochondria quality control, thereby inhibiting ROS generation and safeguarding macrophages from apoptosis under atherosclerotic conditions (49). Moreover, macrophages can phagocytose oxLDL, bringing about elevated levels of proinflammatory cytokines (50). Interestingly, promoting autophagy in microphages can decrease intracellular lipid accumulation, suppress apoptosis and mitigate the development of atherosgenesis, by removing damaged mitochondria and lowering intracellular ROS levels (51). However, a high-protein diet, which elevates amino acid concentrations in bloodstream and atherosclerotic plaques, has been revealed to stimulate macrophage mTOR pathway, thereby inhibiting mitophagy (52). This results in the accumulation of dysfunctional mitochondria and triggers lipid-dependent cell death in macrophages, exacerbating AS. In conclusion, lipid dysbolism, inflammation and other factors play pivotal roles in impairing mitophagy in macrophages. Mitophagy serves a protective function against AS via clearing damaged mitochondria, inhibiting ROS production, suppressing NLRP3 activation, and modulating energy metabolism in macrophages.

#### 4.2.3 Potential therapies targeted mitophagy

In regard to treatment for AS, multiple natural and auxiliary drugs targeting mitophagy have been extensively explored. An animal experiment with a strong citation burst from 2023 onwards, highlighted the continued focus on the protective effects and mechanisms of melatonin in this domain. Melatonin has been reported to impede the development of AS by restraining NLRP3 inflammasome activation and the secretion of inflammation factors (18). Additionally, it induces mitophagy through the Sirt3/FOXO3/Parkin pathway, effectively attenuating mitochondrial ROS production. Another study demonstrated that melatonin may activate mitophagy to alleviate oxidative stress and apoptosis (53). In atherosclerotic regions, melatonin acts as a promising anti-inflammatory agent by modulating mitophagy in macrophages (11). Furthermore, with the aid of nanomedicine and drug delivery systems, melatonin, accompanied by other mitophagy regulators, can be administered to macrophages to achieve enhanced therapeutic effects in alleviating AS.

As for other drugs involved in mitophagy and AS, including pitavastatin (54), rivaroxaban and aspirin (55), dexmedetomidine, liraglutide and coenzyme Q10 (13), have been reported to enhance mitophagy, exerting anti-inflammatory effects and providing cellular protection.

Some natural compounds have been identified as modulators of mitophagy in AS. Gypenoside A, a lipid-selenium binding drug known for its anti-atherosclerotic properties, has been described to mitigate mitophagy dysfunction and mitochondrial dynamics disorder in mice, subsequently restraining mitochondrial pathway-induced necroptosis (56). This regulatory process may occur via NDUFS4-TMBIM6-VDAC1 axis, implying it as a potential therapeutic target. In addition, compounds such as resveratrol, 13-methylberberine, puerarin, scutellarin and salvianolic acid B have been found to promote mitophagy, mitigate endothelial dysfunction and protect ECs.

Several traditional Chinese medicine (TCM) formulas have been reported to enhance mitophagy and alleviate AS. A study elucidated that the Chinese herbal compound Xinmaikang exerted anti-atherosclerotic effect through regulating PINK1/Parkin-induced mitophagy in macrophage (57). Yi Mai granule, compromising two classic Chinese medicine formulas, stimulated mitophagy against atherosclerosis possibly through PINK1-Mfn2-Parkin pathway. This formula also modulated proinflammatory factors, vasoconstrictor cytokines and blood lipid levels (58). Buyang Huanwu Decoction exerts an anti-AS effect through modulating macrophage mitophagy in AS plaques and the TLR4-NF-κB-NLRP3 inflammatory pathway, thereby contributing to stabilization and improvement of AS plaques in mice (59). Notably, multiple studies have found that TCM has anti-atherosclerotic effects via increasing mitophagy (60), representing a promising candidate for future therapeutic interventions. Key compounds in modulating mitophagy in AS are displayed in Table 4.

**Table 4:** Key compounds in modulating mitophagy in AS.

Compound		Mechanism	Model	Observed effect	Reference
Drug	Melatonin	Activates AMPK-OPA1 axis and AMPK-TFEB-Parkin pathway, suppresses NLRP3 inflammasome	VSMC, HUVEC, flap model rat	Enhances mitophagy, inhibits oxidative stress and apoptosis, decreases plaque area	(11, 18, 53)
	Pitavastatin	Activates a calcium-dependent CAMK1-PINK1 pathway	EPC, ApoE−/− mice	Enhances mitophagy, promotes EPC proliferation and vascular re-endothelialization, reduces oxidative stress	(54)
	Rivaroxaban + Aspirin	Increases PINK1/Parkin, reduces ROS production	HCAEC	Enhances mitophagy, inhibits ROS production, protects ECs	(55)
Chinese herbal compound	Xinmaikang (XMK)	Mediates PINK1/Parkin pathway	ApoE−/− mice, RAW264.7	Enhances mitophagy, reduces ROS and lipid levels, reduces atherosclerotic plaque area	(57)
	Yi Mai granule (YMG)	Regulates miRNA-125a-5p, which in turn modulates the PINK1-Mfn2-Parkin pathway	HUVEC, Sprague–Dawley rats	Enhances mitophagy, reduces atherosclerotic plaque area and lesion formation, decreases lipid levels	(58)
	Buyang Huanwu Decoction	Inhibits TLR4/NF-κB/NLRP3 inflammatory pathway	ApoE−/− mice	Stabilizes mitophagy, modulates the gut microbiota, exerts anti-AS effects	(59)

HUVEC, human umbilical vein endothelial cell; EPC, endothelial progenitor cell; HCAEC, human coronary artery endothelial cell.

A range of pharmacological agents that modulate mitophagy in the context of AS have been explored. Nevertheless, further animal experiment and clinical trials are necessary to validate their effectiveness and safety. As of now, there is still a lack of effective and safe therapies for AS specifically targeting mitochondria.

## 5 Limitations

Several limitations must be acknowledged. Firstly, this research incorporated WOSCC data solely, focusing on English-language literatures, which could possibly bias the findings. However, as a most commonly used database when conducting bibliometric analysis, WOSCC covers most of the information. Secondly, current mainstream bibliometric analysis methods are susceptible to citation lag, which may confound the interpretation of short-term data. However, we included data spanning two decades (2004–2024) and performed citation burst analysis, helping to mitigate the analytical biases. To tackle these limitations, future studies are expected to enlarge the range of data acquisition and conduct more comprehensive analysis, providing greater support for researchers. Nonetheless, this study remains valuable for readers concerned about the current situation, hotspots, and forefronts in the domain of mitophagy in AS.

## 6 Conclusion

Over the past few decades, research on the mechanisms of mitophagy in AS has grown significantly, demonstrating its substantial potential and value. This study presents key insights and solid rationales for future advancements. Notably, China and the USA take the lead in this domain. Through analysis of the key articles, it becomes evident that dysfunctional mitophagy is linked to various factors, such as ROS and abnormality in lipid metabolism, with mitochondrial DNA mutations and mitochondrial dynamics alternation playing crucial roles. These factors conduce to impairment of ECs, as well as proliferation and phenotype transformation of VSMCs, thus participating in mechanisms of atherogenesis.

Future research should prioritize, key areas: molecules including NF κB, NLRP3 inflammasome, mitochondrial DNA; pathological process including oxidative stress, mitochondrial dysfunction, mitochondrial dynamics. Investigations on mechanisms of mitophagy and AS in VSMCs, ECs and macrophages also provide valuable insights and draw considerable attention. This study outlines the current research landscape on mitophagy in AS, identifies key research areas, and highlights future hotspots. Continued exploration of mitophagy mechanisms in AS will deepen our understanding and improve management strategies.

## Data Availability

The raw data supporting the conclusions of this article will be made available by the authors, without undue reservation.
