# Supplementary material for: Bibliometric analysis of research hotspots and emerging trends in mitophagy and atherosclerosis (2004–2024)
Source: Front Med (Lausanne). 2025 Jul 25;12:1621079. doi: 10.3389/fmed.2025.1621079 (PMC12331740; doi:10.3389/fmed.2025.1621079)
Supplement: Supplementary file 1 [file Data_Sheet_1.docx]

Supplementary Material

## Supplementary Table

Supplementary Table 1. Specific retrieval strategy.

| Step | Results | Searches |
| --- | --- | --- |
| #1 | 495 | (TS= (Mitophagy) OR TS= (Mitochondrial autophagy) OR TS= (Mitochondrial degradation) OR TS= (Mitochondrial quality control) OR TS= (Mitochondrial clearance) OR TS= (Mitochondrial removal) OR TS= (Clearance of mitochondria) OR TS= (Clearance of mitochondrion) OR TS= (Removal of mitochondria) OR TS= (Removal of mitochondrion) OR TS= (Mitochondria-associated degradation)) AND (TS = (Arteriosclerosis) OR TS= (Fibroatheroma) OR TS= (Fibroatheromas) OR TS= (Arterial Fatty Streak) OR TS= (Arterial Fatty Streaks) OR TS= (Atherosclerotic Plaques) OR TS= (Atherosclerotic Plaque) OR TS= (Atheroma) OR TS= (Atheromas) OR TS= (Atheromatous Plaques) OR TS= (Atheromatous Plaque) OR TS= (Atherosclerosis)) |
| #2 | 456 | Refined by PUBLICATION YEARS: (2004-2024) |
| #3 | 440 | Refined by DOCUMENT TYPES: (ARTICLE OR REVIEW ARTICLE) |
| #4 | 439 | Refined by LANGUAGES: (ENGLISH) |

Supplementary Table 2. Publication counts and confidence intervals from 2004 to 2024.

| Year | Counts | 95%CI | Year | Counts | 95%CI |
| --- | --- | --- | --- | --- | --- |
| 2004 | 6 | (1.67, 3.19) | 2015 | 13 | (13.39, 17.26) |
| 2005 | 3 | (2.02, 3.71) | 2016 | 14 | (16.09, 20.23) |
| 2006 | 2 | (2.45, 4.32) | 2017 | 23 | (19.30, 23.77) |
| 2007 | 3 | (2.96, 5.03) | 2018 | 23 | (23.07, 28.01) |
| 2008 | 2 | (3.58, 5.86) | 2019 | 24 | (27.48, 33.14) |
| 2009 | 9 | (4.33, 6.83) | 2020 | 46 | (32.58, 39.38) |
| 2010 | 4 | (5.23, 7.96) | 2021 | 59 | (38.46, 46.99) |
| 2011 | 6 | (6.32, 9.28) | 2022 | 54 | (45.25, 56.27) |
| 2012 | 6 | (7.64, 10.82) | 2023 | 54 | (53.08, 67.58) |
| 2013 | 11 | (9.22, 12.63) | 2024 | 61 | (62.14, 81.32) |
| 2014 | 16 | (11.12, 14.76) |  |  |  |

CI, Confidence interval.

## Supplementary Figures


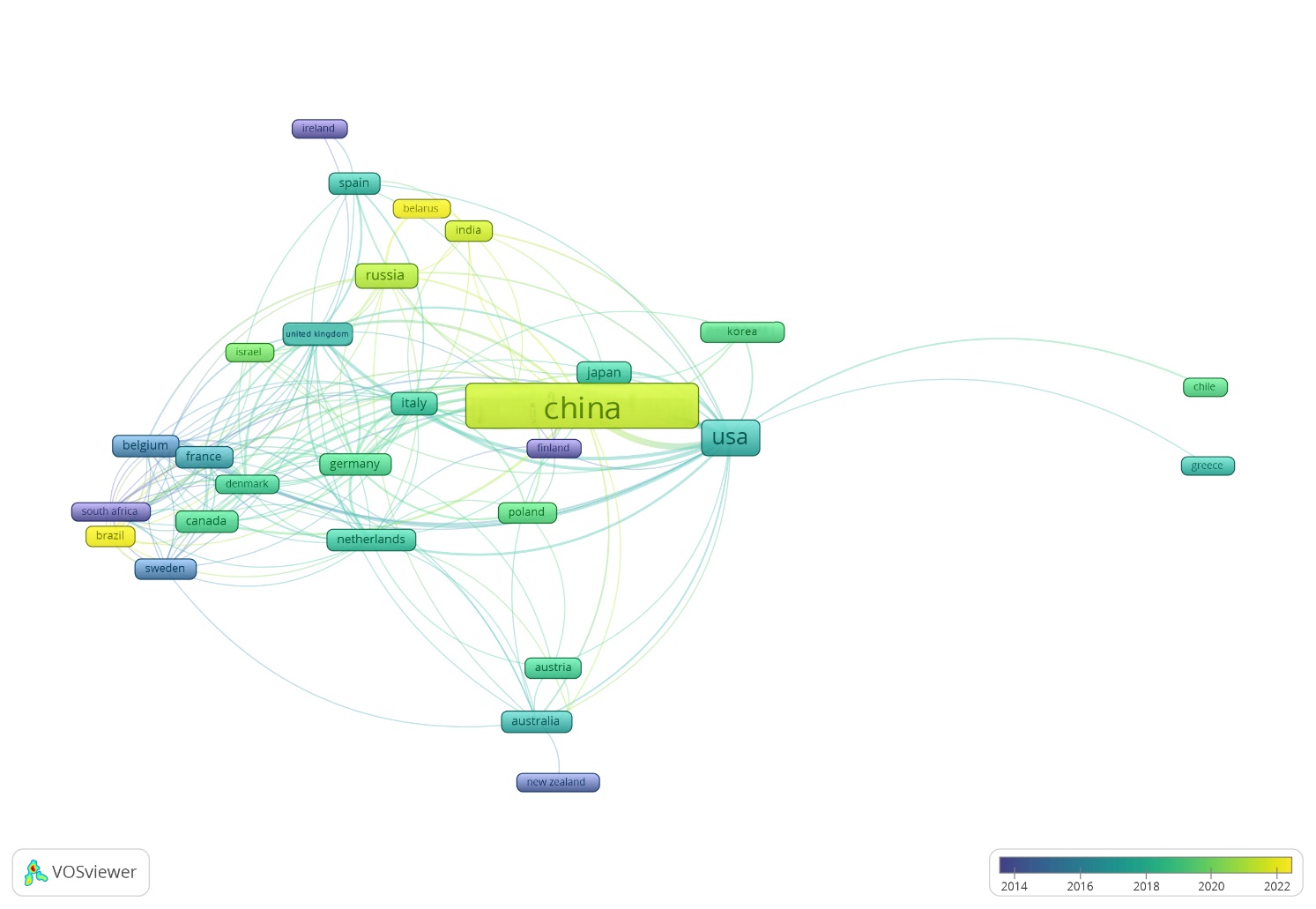
**Supplementary Figure 1.** The co-occurrence network map of countries/regions. The node size indicates the publication volume, and the cooperation is exhibited as links between nodes. The thicker the line is, the closer the cooperation is.

**
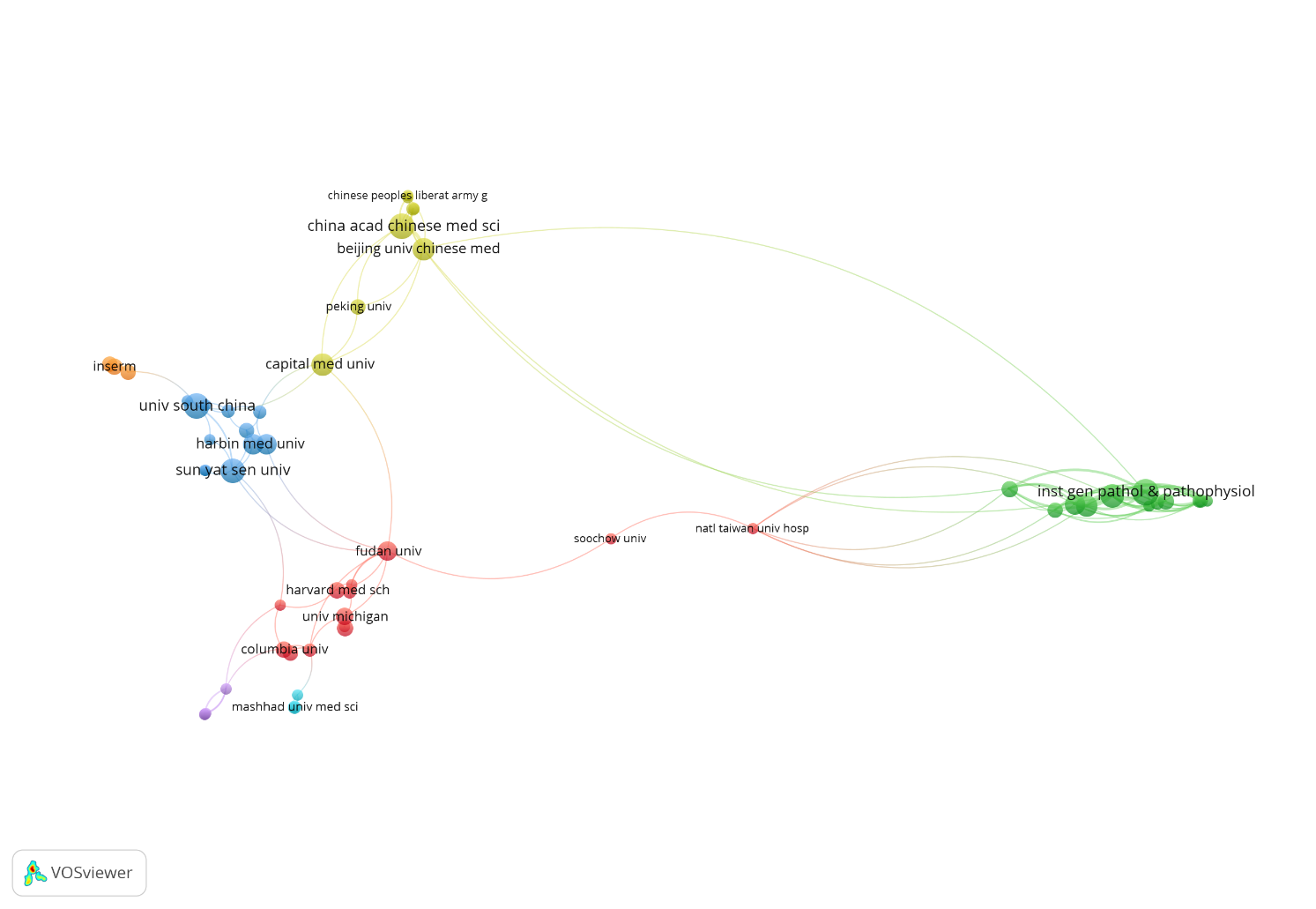
**

**Supplementary Figure 2.** Institutions co-occurrence map.


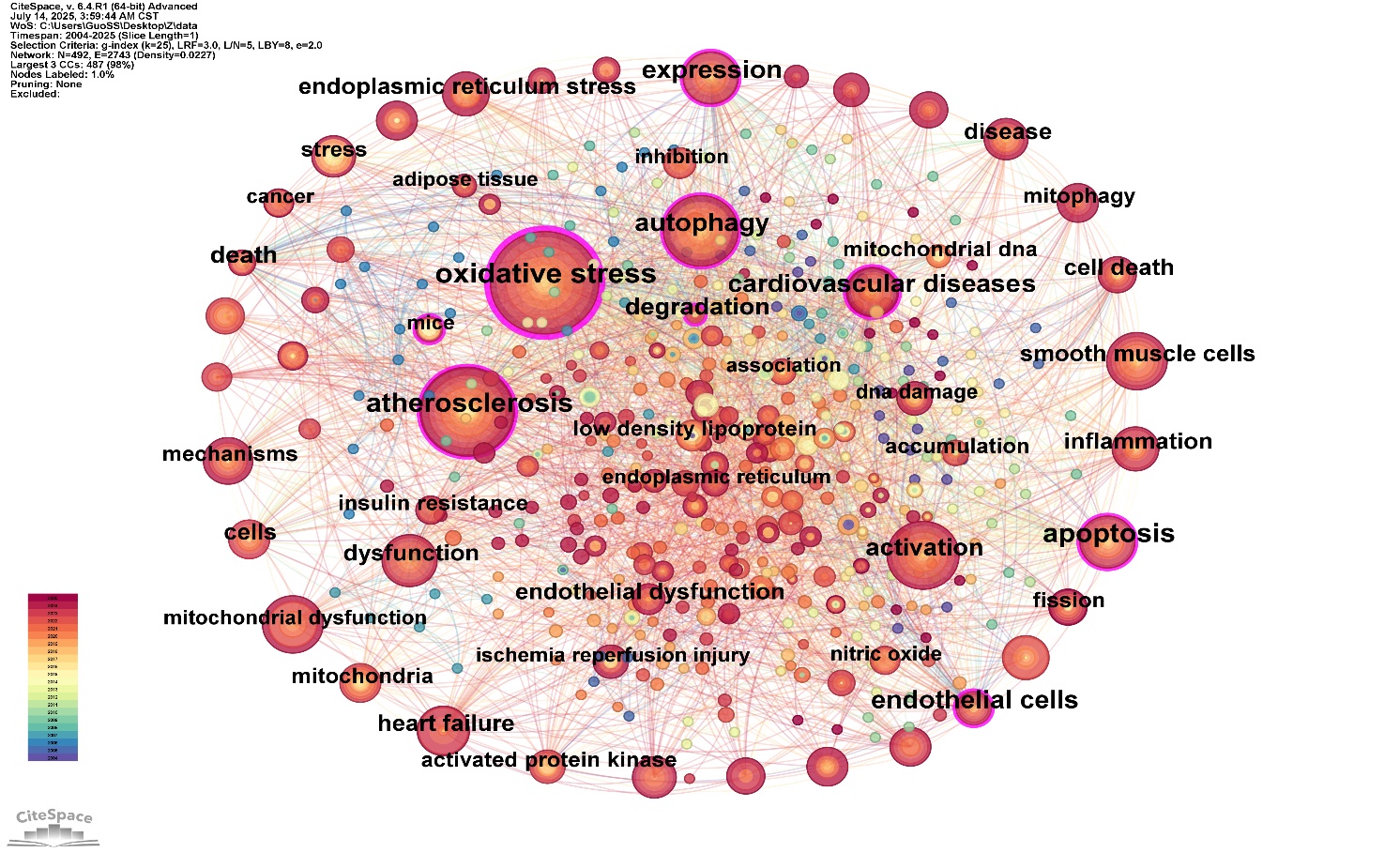


**Supplementary Figure 3.** Keyword co-occurrence network map.


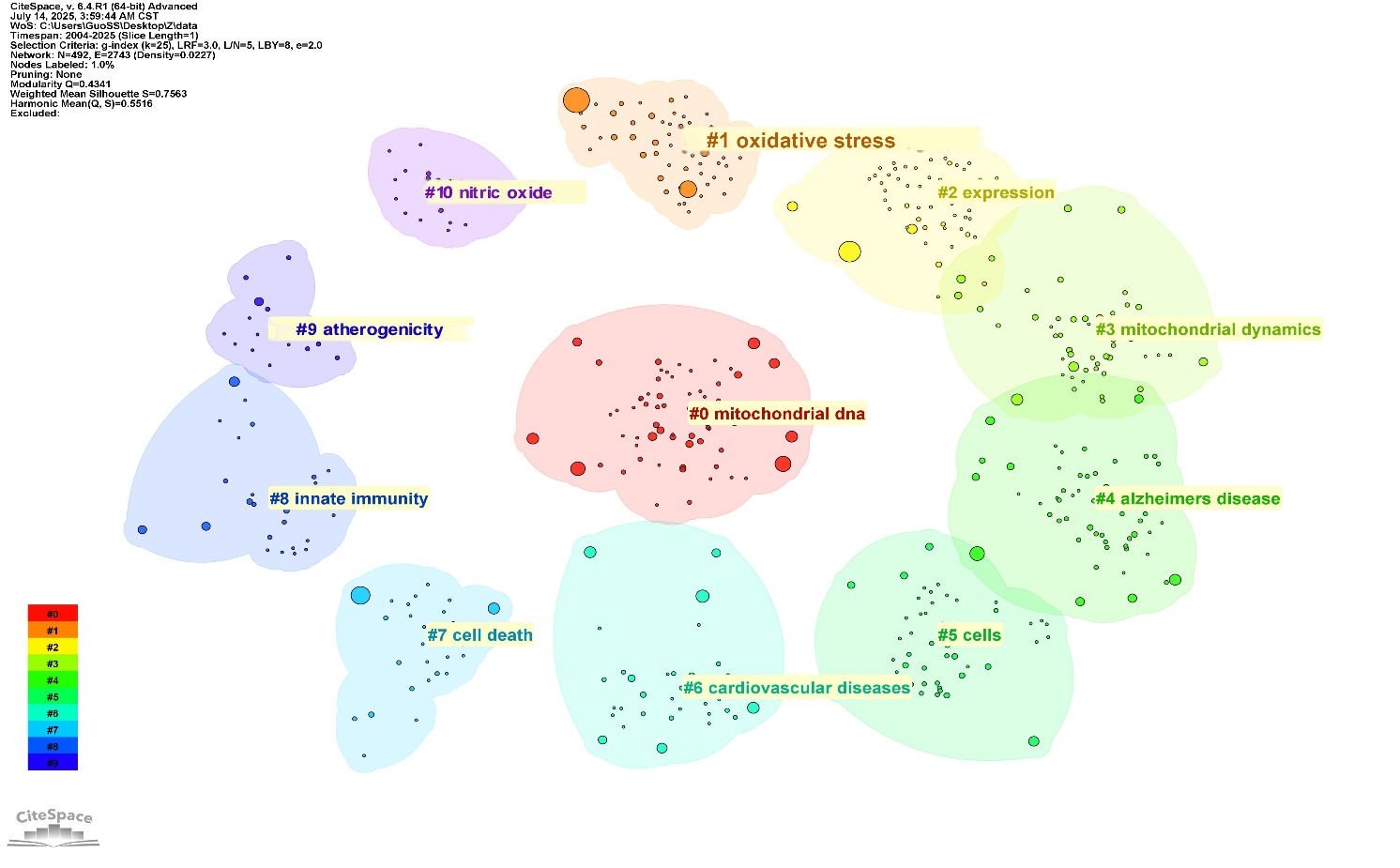


**Supplementary Figure 4.** Cluster analysis of keywords.


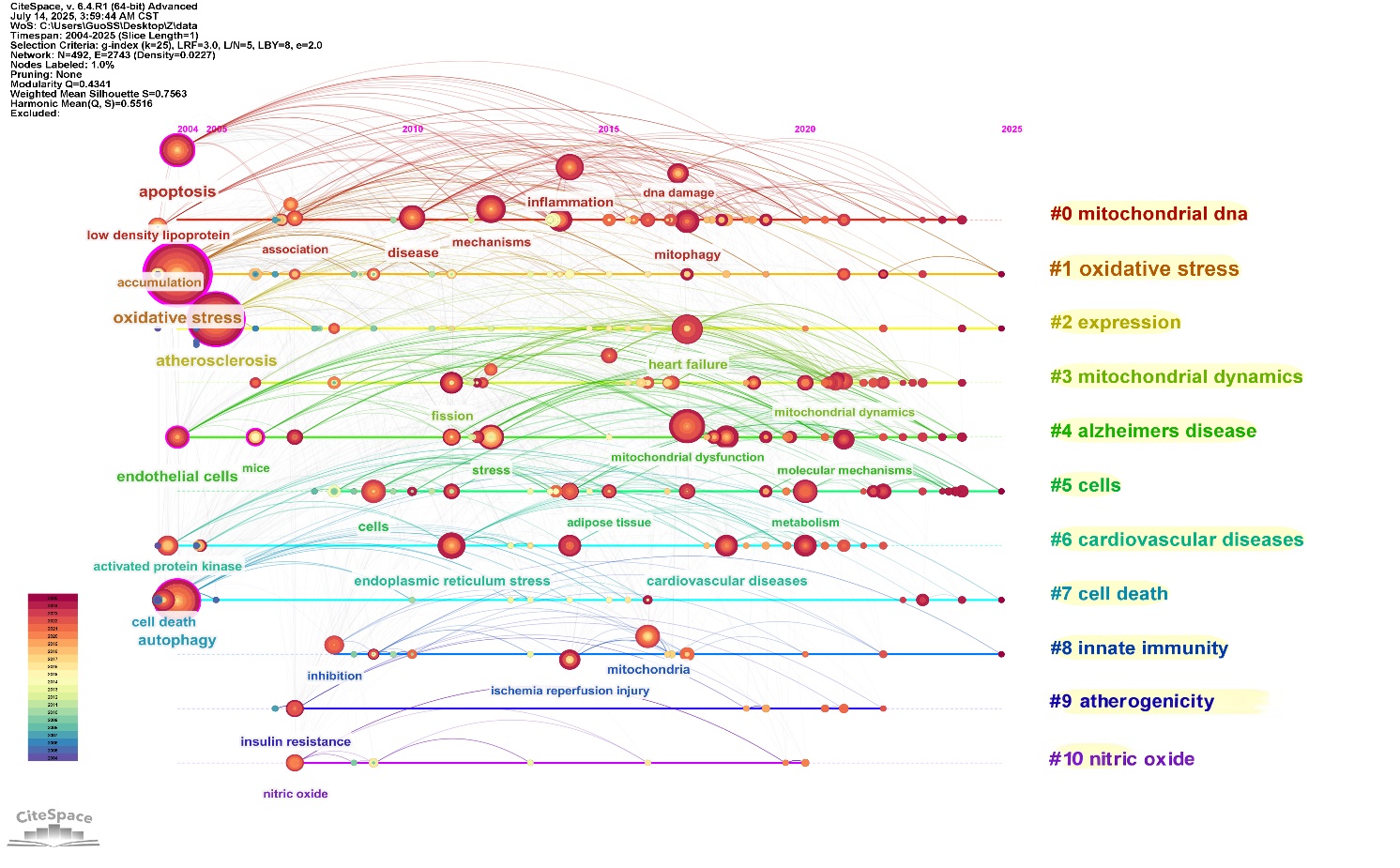


**Supplementary Figure 5.** Timeline distribution of the top 9 clusters.
